# Ensuring Sustainability in Pharmaceutical Care: A Retrospective Analysis of Administrative Databases on the Utilization, Costs, and Switching Patterns of Biological Therapies in the Agency for Health Protection of the Metropolitan Area of Milan

**DOI:** 10.3390/ph18040482

**Published:** 2025-03-27

**Authors:** Renata Maria Bianca Langfelder, Roberto Langella, Cinzia D’Angelo, Claudia Panico, Sarah Cattaneo

**Affiliations:** Pharmaceutical Department, Health Protection Agency of the Metropolitan City of Milan, 20122 Milan, Italy; rlangfelder@ats-milano.it (R.M.B.L.); cidangelo@ats-milano.it (C.D.); cpanico@ats-milano.it (C.P.); scattaneo@ats-milano.it (S.C.)

**Keywords:** biological drugs, biosimilars, originator, cost-containment strategy, therapeutic, sustainability, switching

## Abstract

**Background**: Biosimilars represent a fundamental advancement in global healthcare, offering significant cost containment while maintaining both therapeutic efficacy and safety in the management of chronic diseases. The cost savings generated by adopting biosimilars could be reinvested to foster innovation in the healthcare sector and enhance patient access to advanced therapies. **Methods**: A comprehensive analysis was conducted within an Italian healthcare organization which, through its hospital network, serves over 3.5 million individuals. Usage patterns, expenditure, and patient coverage for the principal biosimilar agents across various therapeutic areas were examined. Data were extracted from institutional registries, and a year-over-year comparison from 2022 to 2024 was performed to evaluate trends in consumption, biosimilar adoption among treatment-naïve patients, incurred costs, potential and actual savings, as well as therapeutic switching profiles. **Results**: The analysis revealed a marked shift towards biosimilar formulations for the majority of the evaluated biological agents between 2022 and 2024. However, for certain active substances, a reduced market penetration of biosimilars was observed, and critical issues persist that will necessitate future interventions. **Conclusions**: The results demonstrate a consistent upward trajectory in biosimilar adoption, underscoring significant progress toward their integration into routine clinical practice—a transition that has generated substantial savings over the three-year period considered. Assuming a complete transition to biosimilars, the cumulative potential savings over the three-year period would amount to EUR 7,172,372.99 in 2022, EUR 6,209,289.05 in 2023, and EUR 23,536,824.05 in 2024. This trend aligns with strategic objectives to enhance the sustainability of the Italian National Health Service (SSN) through optimized resource allocation and improved patient access.

## 1. Introduction

Biological drugs, produced using living cells or developed through advanced biotechnological engineering techniques, represent a cornerstone of contemporary pharmacotherapy due to their capacity to act with high specificity on molecular targets that play pivotal roles in the pathogenesis of many complex diseases. These highly complex biological agents—including hormones, growth factors, cytokines, interferons, vaccines, monoclonal antibodies, and fusion proteins—have radically transformed the therapeutic landscape of numerous high-impact conditions [1].

Through highly selective mechanisms of action aimed at specific cellular and molecular pathways, their use has revolutionized the clinical management of diseases such as rheumatoid arthritis, ankylosing spondylitis, psoriasis, ulcerative colitis, Crohn’s disease, and various neoplasms, resulting in significant improvements in both therapeutic outcomes and patients’ quality of life [2]. Despite their undeniable clinical benefits, biological drugs pose a significant challenge to healthcare systems, primarily in terms of economic sustainability. These therapies rank among the most expensive pharmacological options, largely due to the complexity inherent in the research, development, and production processes [3]. Unlike chemically synthesized low-molecular-weight drugs, biological drugs require the use of living cells under highly controlled culture conditions, rendering their production not only particularly costly but also susceptible to variations in manufacturing protocols. Consequently, ensuring access to such innovative treatments within the budget constraints of public healthcare systems remains an urgent issue, especially in countries like Italy, where the universal healthcare model strives to guarantee an equitable distribution of therapeutic resources [4].

The introduction of biosimilars constitutes a key strategy to reduce the financial impact associated with biological therapies. Biosimilars are biological medicinal products developed to faithfully replicate the same active substance as that of a reference product—whose patent protection has expired—thereby ensuring a quality, safety, and efficacy profile that is substantially analogous to the originator. This outcome is achieved through rigorous comparability studies conducted in accordance with standards established by regulatory authorities, including the European Medicines Agency (EMA). Such studies encompass advanced analytical evaluations, non-clinical investigations, and clinical trials designed to demonstrate “biosimilarity” [5,6].

Recently, the EMA and the Heads of Medicines Agencies (HMA) issued a joint statement confirming the interchangeability of biosimilars approved within the European Union (EU) with their corresponding reference products, as well as the possibility of substituting one biosimilar for another of the same reference medicinal product [7]. This common position—already implemented in several Member States—aims to harmonize the EU-wide approach and to provide clear guidance to healthcare professionals, thereby enhancing patient access to essential biological therapies. According to the EMA and HMA, once approved in the EU, a biosimilar may be used in place of the originator (or vice versa) and may similarly be substituted with another biosimilar containing the same active ingredient. However, it remains within the purview of individual Member States to decide whether to permit automatic substitution at the pharmacy level without prior consultation with the prescribing physician [8,9].

In Italy, the Italian Medicines Agency (AIFA) has clarified that biosimilars cannot be regarded “sic et simpliciter” on par with generic drugs, emphasizing that, although the risk–benefit profile of biosimilars is comparable to that of the reference originators, the responsibility for selecting the most appropriate treatment for each patient rests with the prescribing physician. This decision must take into account, among other factors, specific clinical needs, the sustainability of the healthcare system, and proper communication to patients regarding the use of biosimilars. AIFA thus recognizes interchangeability between biosimilars and bio-originators for both naïve patients and those already in treatment, while specifying that any substitution should be based on clinical evaluation and cannot be performed automatically at the pharmacy level [10].

This stance, while in line with the common European approach [11], maintains a measure of caution regarding automatic substitution, reflecting the need for case-by-case clinical assessment. Beyond the clinical arena, biosimilars offer significant and well-documented economic benefits. With prices reduced by up to 30% compared to the originators, the use of biosimilars enables healthcare systems to achieve substantial cost savings. According to OsMed-AIFA data [12] and related analyses, in Italy the potential savings derived from substituting originators with biosimilars can exceed EUR 300 million per year, depending on the molecules involved and the actual market penetration achieved [13].

Within the Italian context, public healthcare facilities procure drugs exclusively through competitive bidding or framework agreements—a mechanism that incentivizes cost containment while ensuring the availability of high-quality treatments. The integration of biosimilars into this procurement model has had transformative effects, providing healthcare operators with a sustainable pathway to broaden patient access without compromising clinical outcomes. Despite the scientific validation of safety and efficacy standards and the clear economic advantages, the adoption of biosimilars in Italy remains heterogeneous both across regional policies and among the various therapeutic areas addressed by these drugs [14].

Partial resistance persists, particularly with respect to high-cost biologics, as both prescribers and patients often exhibit a preference for originators—frequently driven by product familiarity or subjective biases regarding an assumed superior efficacy of the originators. Literature analyses [15] confirm that such perceptual biases slow the adoption of biosimilars, underscoring the need for educational initiatives and transparent communication.

**Figure 1:**
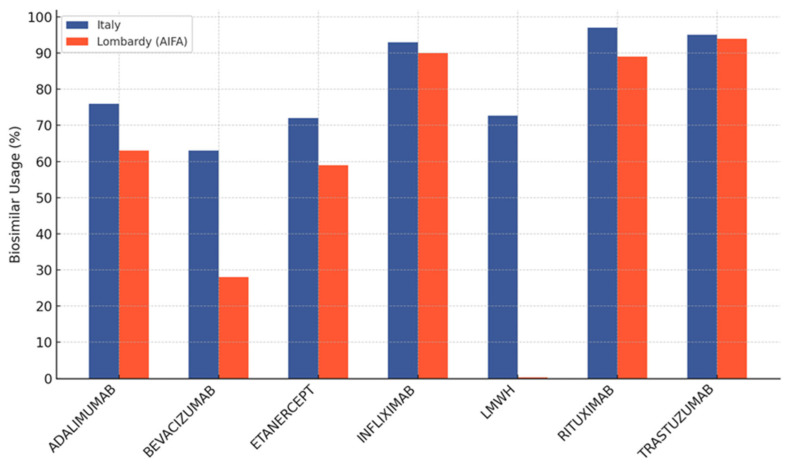
Percentage of biosimilar utilization with the highest volume/cost impact, comparing the national trend and that in Lombardy for the year 2021. The data are extracted from the December 2021 Report of the Periodic Monitoring on Biosimilar Utilization published by the Italian Medicines Agency (AIFA).

**Figure 2:**
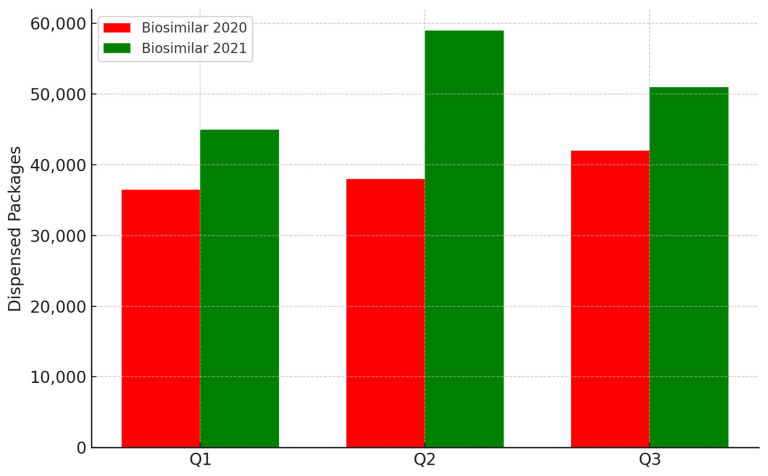
Dispensed packages of biosimilars in Lombardy: comparison between the years 2020 and 2021. The data are extracted from the December 2021 Report of the Periodic Monitoring on Biosimilar Utilization published by the Italian Medicines Agency (AIFA).

Over time, these challenges have highlighted the necessity of launching interventions at both the health policy and educational levels, thereby complementing existing communication strategies promoted by the pharmaceutical industry. The objective is to reassure clinicians and patients regarding the therapeutic equivalence of biosimilars and originators, and to encourage informed uptake of these therapeutic options. The Lombardy Region—the fourth-largest in Italy and the most populous, with approximately 10 million inhabitants—has implemented policies aimed at increasing the use of biosimilars in clinical practice. According to periodic AIFA reports, as of December 2021, Lombardy was below the national average in the utilization of several biosimilar molecules (Figure 1 and Figure 2) [16].

For instance, for adalimumab, the regional incidence percentage (in terms of packages consumed) was 76.8% compared to a national figure of 81.9%; for bevacizumab, the Lombardy utilization rate was 45.9% versus 88.6% nationally; and for low-molecular-weight heparins, the regional figure (0.3%) was markedly lower than the national average (72.7%). In response to these findings, starting in 2022 the Lombardy Region established specific objectives to increase the penetration of biosimilars in all public and accredited private healthcare facilities operating within the region, through the Health Protection Agencies (ATS—*Agenzie di Tutela della Salute*). In particular, the General Welfare Directorate resumed the production of monitoring reports related to the use of certain drug classes (including biosimilars), thereby providing a tool not only for analysis but also for planning. Key elements of this strategy include the monitoring of biological molecules with expired patents, the evaluation of the impact of biosimilars on the efficiency of direct procurement, and the promotion of an integrated model between intra- and extra-hospital settings [17]. For 2024, the General Welfare Directorate has set an objective for the ATS to promote the biosimilar as the most economically sustainable therapeutic option compared to the originator [18]. Strategic actions include raising awareness among hospital prescribers to consider biosimilars as the first therapeutic choice when clinically appropriate, promoting the use of biosimilars both in hospital settings and in community care, organizing joint training and communication initiatives with healthcare service providers to support professionals in the correct interpretation of efficacy and safety data, and conducting periodic analyses (*File F*) of biosimilar consumption to identify critical therapeutic areas with improvement potential. The ATS of the Metropolitan City of Milan—the largest in the region in terms of population (approximately 3.5 million inhabitants) and number of healthcare facilities—manages the pharmaceutical expenditure of nine public Territorial Healthcare and Social Agencies (ASST—*Aziende Socio Sanitarie Territoriali*), three public Scientific Institutes for Research, Hospitalization and Healthcare (IRCCS), as well as over ten accredited private IRCCSs and numerous accredited private care facilities. To meet regional objectives, the Pharmaceutical Department of the ATS of Milan has, over the past three years, initiated intensive monitoring of usage patterns and the financial impact of both originator and biosimilar biological drugs. In parallel, the ATS of Milan has developed a training program aimed at healthcare professionals (including general practitioners, hospital and community specialists, and pharmacists) and patient education initiatives designed to disseminate scientific knowledge regarding the equivalence in efficacy and safety of originators and biosimilars. This program promotes trust among professionals and patients in biosimilar drugs through transparent communication and multidisciplinary exchanges (conferences, workshops, distance learning training), and it provides practical tools to support clinical decision-making (regional guidelines, cost comparison tables, real-world evidence). At the conclusion of the three-year period (2022–2024), the analysis conducted by the ATS of Milan revealed a substantial increase in the use of biosimilars. In 2022, data already indicated that the complete substitution of originators with the corresponding biosimilars could have generated significant savings—approximately EUR 2.63 million for adalimumab biosimilars and around EUR 2.48 million for etanercept biosimilars. In view of the continued reliance on originator drugs, further training and awareness initiatives were implemented, contributing to increased biosimilar penetration rates, as reflected in the data analysis at the end of 2024. Analysis of consumption trends and costs, together with studies on management practices for naïve patients and strategies for therapeutic switching, confirms that healthcare systems can achieve substantial savings by consciously promoting the use of biosimilars. Nevertheless, cultural and relational obstacles persist, manifesting as hesitancy among physicians and biases among patients. The systematic adoption of biosimilars not only reduces the economic burden of high-cost biological therapies but also aligns with the equity objectives of universal healthcare systems, ensuring access to high-quality care without compromising the sustainability of public expenditure. The Italian experience with biosimilars—and in particular that developed within the ATS of the Metropolitan City of Milan, which operates in a demographically and organizationally complex territory comparable to certain medium-sized European countries such as Sweden, Portugal, and Hungary—demonstrates that the implementation of appropriate regulatory frameworks, effective purchasing and pricing strategies, as well as targeted educational and informational interventions are crucial for promoting the use of the medicinal product with the best cost profile while maintaining equivalent efficacy and safety. These findings serve as a valuable reference for other healthcare systems aiming to combine expanded therapeutic access with economic sustainability. The primary objectives of this study were to assess biosimilar adoption trends across multiple therapeutic areas, quantify the economic impact of biosimilar integration compared to originator products, and analyze therapeutic switching patterns in a large Italian healthcare setting. Additionally, the study aimed to identify key barriers influencing biosimilar penetration, with the hypothesis that enhanced adoption would result in substantial economic savings and improved healthcare sustainability.

## 2. Results

### 2.1. Drug Utilization

The analysis of data from the 2022–2024 period highlighted variable levels of biosimilar adoption across the 20 therapeutic classes of off-patent drugs examined in this study, within a context characterized by a complex interplay of clinical, economic, and market factors (Table 1). The results demonstrated a progressive increase in the use of biosimilars during the observation period. Overall, of the 56,406 patients analyzed, 27,535 (48.8%) were treated with biosimilars, while 28,869 (51.2%) received the originator product. However, this distribution exhibits significant differences depending on the specific therapeutic area and the administration setting.

#### 2.1.1. Insulin Therapies

Within the realm of insulin therapies, there is a marked predominance in the use of originator products. Lispro insulin was almost exclusively administered as the originator, with a modest increase in the use of biosimilars observed in 2024 (1.6%). Similarly, aspart insulin was used exclusively as the originator (100% of administrations), whereas Glargine insulin showed a progressive reduction in the biosimilar share, declining from 36.5% in 2022 to 17.5% in 2024. It is important to note that these drugs are predominantly distributed through community pharmacies. Consequently, the analysis—limited to distribution in hospital pharmacies—is not fully representative of the actual territorial utilization of biosimilars for this category of drugs. Furthermore, public healthcare institutions procure these drugs through regional tenders which, over the examined three-year period, have predominantly favored originator products for the supply of off-patent drugs.

#### 2.1.2. Anticoagulants and Erythropoietins

Regarding anticoagulants and erythropoietins, the uptake of biosimilars has been modest. In particular, sodium enoxaparin registered a biosimilar market share of 4.9% in 2022—a figure that increased significantly to 57.4% in 2024. This evolution was promoted by awareness campaigns targeting prescribing physicians, aimed at encouraging the use of medications with a more favorable economic profile while ensuring clinical efficacy equivalence, both at the regional level and through the intervention of ATS Milan. It is important to note that, for this medication, the procurement process under a framework agreement has involved both the originator product and biosimilar molecules since 2019. However, at the hospital level, the competitive pricing of the originator has strongly incentivized its use. In light of the relatively low volumes of sodium enoxaparin used in hospitals, the drug exhibits substantially higher consumption and expenditure in the community setting. The introduction of the originator product, within a continuum of care bridging hospital and community, has also led to its increased use in the community—dispensed by local pharmacies—with costs markedly higher than those expected in the hospital context. In 2022, the evaluations conducted by the III Commission for “Health and Social Policies” of the Lombardy Region assessed the territorial impact of sodium enoxaparin. According to the Commission, directly transferring the expenditure associated with the originator to a biosimilar consumption model would have resulted in savings of EUR 8.7 million [19]. Based on these assessments, targeted policies were implemented to favor the medication with the optimal cost profile, taking into account its potential territorial impact. The measures adopted in this regard enabled a substantial increase in the uptake of the biosimilar by the end of the examined three-year period. The experience with sodium enoxaparin demonstrates that implementing biosimilars in hospitals is a crucial strategy for also promoting the introduction of the most cost-effective drug at the community level, thereby contributing to more sustainable management of healthcare resources—especially in contexts where dispensation volumes are particularly high and the costs of originator products exert a greater impact on the budget. Within the field of erythropoietins, data indicate a consolidated adoption of biosimilars: epoetin alfa reached a market share of 61.1% in 2024, while epoetin zeta achieved complete replacement of the originator product, maintaining a constant 100% adoption throughout the three-year period. These results suggest that, when robust evidence of efficacy is coupled with economic advantages, the integration of biosimilars into clinical practice can attain very high penetration rates.

#### 2.1.3. Onco-Hematological Drugs and Medications for Autoimmune Diseases

Even in the onco-hematological sector and in autoimmune disorders—both characterized by the use of high-cost biological drugs—there has been a rapid adoption of biosimilars. In particular, trastuzumab maintained a biosimilar market share of 99.5% in 2024; pegfilgrastim increased from 99.9% in 2022 to 100% in 2024; bevacizumab rose from 91.8% to 99.6%; rituximab from 95.3% to 97.9%; and infliximab from 92.6% to 97% over the same period. These high adoption rates result from targeted initiatives aimed at cost containment in high-impact therapies, supported by robust clinical evidence confirming their efficacy and safety, which in turn has fostered their widespread acceptance. For other biological drugs, such as etanercept and adalimumab, significant room for improvement remains. Despite regional awareness campaigns, biosimilar adoption reached 73.8% for etanercept and 86.3% for adalimumab in 2024, compared with penetration levels of 59.2% and 71.8%, respectively, in 2022. Although these figures are relatively high, they underscore the presence of barriers related both to clinician and patient acceptance, thereby impeding a complete transition to biosimilars despite their well-established efficacy and safety profiles, which are comparable to those of the originator products. In addition to issues of prescribing hesitancy, it is noteworthy that during 2022 and 2023, certain production challenges experienced by the manufacturers of biosimilars—who secured the regional tenders—resulted in shortages across distribution channels. This situation led to a reduction in biosimilar penetration, accompanied by a concurrent shift of patients—both treatment-naïve and those already under therapy—back to the originator product. These dynamics underscore the importance of ensuring a robust and continuous supply chain, capable of guaranteeing the constant availability of biosimilars and preventing adoption fluctuations due to supply issues.

#### 2.1.4. Analysis of Prescriptions in Naïve Patients

The analysis of data pertaining to naïve patients—those initiating therapy with specific active ingredients for the first time during the study periods—reveals a variable distribution between biosimilar drugs and originators. A comparison between 2023 and 2024 demonstrates a clear trend toward increased adoption of biosimilars among naïve patients (Table 2).

For the active ingredient adalimumab, there is an observed rise in the use of biosimilars, increasing from 1050 naïve patients in 2023 to 1127 in 2024, while the number of naïve patients treated with the originator decreases from 124 to 46. This trend confirms the growing preference for biosimilars among this patient group. Regarding bevacizumab, the situation remains nearly unchanged, with 510 naïve patients treated with the biosimilar in 2023 compared to 512 in 2024. In contrast, the number of naïve patients initiated on the originator drug falls from nine to three, suggesting a further, albeit marginal, shift toward the biosimilar. In the case of enoxaparin sodium, there is a substantial increase in the number of naïve patients treated with the biosimilar, rising from 779 in 2023 to 2534 in 2024, while those treated with the originator sharply decrease from 5066 to 2356. This indicates a strong trend toward the implementation of the biosimilar formulation. For etanercept, the number of patients treated with the biosimilar increases from 268 in 2023 to 293 in 2024, whereas the number of naïve patients receiving the originator declines from 27 to 16, highlighting a clear preference for the biosimilar alternative. In the case of infliximab, the number of naïve patients treated with the biosimilar shows a slight decrease, falling from 445 in 2023 to 407 in 2024, while the number of patients on the originator remains stable with a modest increase from five to six. This pattern suggests a potential saturation in the transition to the biosimilar. Overall, these data indicate heterogeneity in therapeutic choices among the various active ingredients, likely influenced by factors already highlighted in the drug utilization analysis.

### 2.2. Pharmacoeconomic Evaluation

Analysis of cumulative expenditure over the three-year period reveals significant variability in the costs associated with biosimilars and reference products across various therapeutic areas, reflecting divergent utilization patterns and complex market dynamics (Table 3).

Between 2022 and 2024, overall spending on the analyzed biological drugs decreased from EUR 67,622,993.68 in 2022 to EUR 66,700,275.09 in 2024. The case of enoxaparin sodium illustrates a marked shift toward the use of biosimilars. Specifically, expenditure on the reference product declined from EUR 118,598.38 in 2022 to EUR 108,671.88 in 2023, further decreasing to EUR 51,959.67 in 2024. Concurrently, spending on biosimilars increased from EUR 5649.15 in 2022 to EUR 28,407.28 in 2023, reaching EUR 69,684.12 in 2024. This trend can be directly attributed to initiatives aimed at promoting the adoption of biosimilars, designed to mitigate the impact of the transition from hospital-based care to community-based care. In 2023, rituximab recorded a total expenditure of EUR 2,034,626.49, of which EUR 1,682,269.09 was allocated to biosimilars; the share of spending on biosimilars reached 86.5% in 2024. In the field of insulin therapies, the adoption of biosimilars remained marginal. The entire expenditure for insulin aspart, amounting to EUR 197,055.99 over the three-year period, was incurred for the reference product. Regarding insulin glargine, only a modest expenditure on biosimilars was observed, amounting to EUR 4805.23, which represents 9.1% of the total spending of EUR 52,938.38. The uptake of biosimilars for epoetin alfa exhibited steady growth. Expenditure on biosimilars increased from EUR 189,983.93 (35.1% of EUR 541,819.71) in 2022 to EUR 249,148.68 (43.5% of EUR 572,202.15) in 2023, reaching EUR 342,560.59 (52.5% of EUR 652,077.67) in 2024. Similarly, epoetin zeta completed its transition to exclusive biosimilar use, with expenditures of EUR 167,714.95 in 2022, EUR 175,713.72 in 2023, and EUR 116,483.56 in 2024. The decline observed in 2024 may reflect variations in clinical utilization or procurement strategies. In the realm of chronic and autoimmune diseases, considerable variability was noted in the adoption of biosimilars. Biosimilars of etanercept demonstrated consistent growth, with expenditures increasing from EUR 1,753,021.60 (31.5% of EUR 5,566,827.01) in 2022 to EUR 1,945,103.99 (35.6% of EUR 5,459,289.19) in 2023, and reaching EUR 2,464,617.83 (49.8% of EUR 4,946,987.83) in 2024. In the latter year, spending on biosimilars surpassed that on the reference product, for which expenditure declined from EUR 3,813,805.41 in 2022 to EUR 3,514,185.20 in 2023 and further to EUR 2,482,370.00 in 2024. Similarly, biosimilars of adalimumab exhibited progressive growth. Expenditure on these biosimilars increased from EUR 2,900,790.33 (41.9% of EUR 6,927,268.37) in 2022 to EUR 3,423,767.18 (47.7% of EUR 7,169,585.62) in 2023, reaching EUR 3,698,915.53 in 2024. Concurrently, the share of expenditure on biosimilars surpassed that of the reference product, for which spending decreased from EUR 4,026,478.04 in 2022 to EUR 3,745,818.44 in 2023 and further to EUR 2,446,345.00 in 2024, with biosimilars accounting for 60.2% of the total expenditure (EUR 6,145,260.53).

#### Potential and Actual Savings

**Figure 3:**
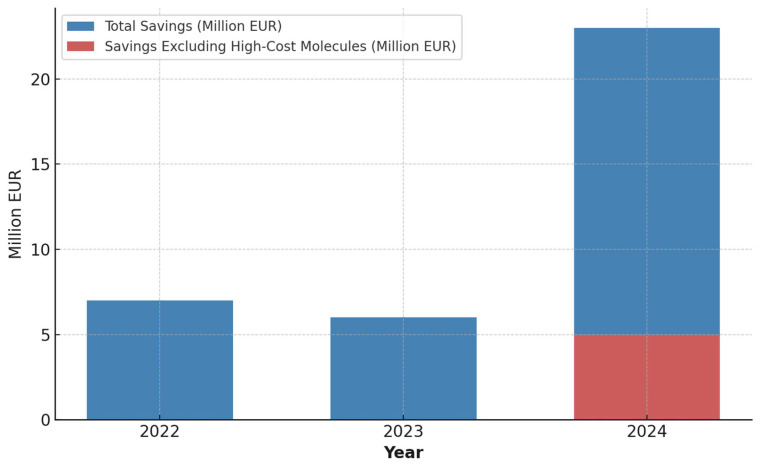
Projected cumulative savings from the transition to biosimilars (2022–2024). The sharp increase in 2024 is due to the market entry of biosimilars for high-cost molecules. When excluding these, savings remain considerable but are more in line with previous years.

Assuming a complete transition to the use of biosimilars, the cumulative potential savings over the three-year period would amount to EUR 7,172,372.99 in 2022, EUR 6,209,289.05 in 2023, and EUR 23,536,824.05 in 2024. The particularly high value observed in 2024 is attributable to the market introduction of biosimilars for high-cost molecules (eculizumab, natalizumab, ranibizumab, tocilizumab, ustekinumab), whose market penetration remains limited. Excluding these five molecules from the calculation, a saving of EUR 4,203,741.23 would still be achieved in 2024 (Figure 3).

These findings are consistent with the progressive adoption of biosimilar drugs in clinical practice. To estimate the savings, the average costs of biosimilars were considered—calculated by relating the total expenditure incurred for these drugs to the actual volume administered. Consequently, the estimated savings should not be regarded as definitive, since biosimilar prices may vary among different hospital facilities. It is also important to consider the additional costs that would have been incurred had the biosimilar utilization percentages from 2022 remained unchanged in 2023 and 2024. In this analysis, the five molecules for which the corresponding biosimilar was introduced to the market after 2022 (eculizumab, natalizumab, ranibizumab, tocilizumab, ustekinumab) were excluded. For methodological consistency, the annual average cost of the originator was used, as its price, too, can vary depending on the type of hospital facility (public or private). Under these premises, the additional costs would have amounted to EUR 1,573,032.28 in 2023 and EUR 3,915,515.18 in 2024, for a total of EUR 5,488,547.47 over the two-year period. It is important to emphasize that, thanks to the promotion, awareness, and public health initiatives adopted by ATS Milano and the Region, these additional costs—exceeding EUR 5 million—were effectively saved. Moreover, based on estimates of a complete transition to the use of biosimilars, the overall potential savings in the coming years could be even greater, with additional savings of several million euros.

### 2.3. Therapeutic Switches

Analysis of switching patterns between biosimilars and originator drugs delineates an evolving scenario. To this end, the number of patients who underwent a switch (from biosimilar to originator, from originator to biosimilar, or multi-switch) or a swap (i.e., the transition between two biosimilars containing the same active substance) was recorded for each year (Table 4).

Over the three-year period, the majority of transitions (54.1%, corresponding to 3389 of 6263 total switches) occurred from one biosimilar to another biosimilar of the same active substance (swap). This finding is particularly relevant in high-cost therapies, such as those involving adalimumab, infliximab, and rituximab, and reflects the growing confidence among healthcare professionals in the therapeutic equivalence of biosimilars, supported by robust regulatory frameworks and competitive tendering processes that favor framework agreements—thus enabling the immediate procurement of an alternative biosimilar of the same molecule in the event of shortages or unavailability of other tendered products [20,21,22,23]. During the same period, transitions from biosimilar to originator were relatively limited, with 33 cases in 2022, 80 in 2023, and 71 in 2024. In certain contexts, these reverse switches were triggered by temporary market shortages of biosimilar drugs (as in the case of etanercept in 2023). In some instances, the prescriber opted for the originator rather than an additional biosimilar, also due to market dynamics in which, in some situations, the originator secures the regional tender by offering the most advantageous price. Transitions from originator to biosimilar exhibited an upward trend over the three-year period, rising from 165 cases in 2022 to 239 cases in 2023, and reaching 1423 cases in 2024. To further examine this significant increase, we performed a pairwise analysis by constructing a 2 × 2 contingency table that compared the frequency of originator-to-biosimilar transitions versus all other transitions between 2023 and 2024. The resulting chi-square test confirmed that the frequency observed in 2024 was significantly higher (*p* < 0.001), thereby reinforcing the effectiveness of policies designed to incentivize the adoption of biosimilars, as well as educational initiatives targeted at both prescribers and hospital pharmacists regarding the benefits of interchangeability between biosimilars and originators. Nevertheless, the heterogeneity observed across different therapeutic areas indicates that barriers persist in some contexts, where clinicians remain reluctant to substitute an originator with a biosimilar in patients long stabilized on the original therapy. Particularly noteworthy are the so-called multi-switches, i.e., sequences of multiple transitions (from biosimilar to originator, then back to biosimilar, or vice versa), which remained relatively stable over the three-year period, with 312 cases in 2022, 289 in 2023, and 262 in 2024. These data are consistent with the existing literature, which indicates that most patients continue to maintain clinical stability even after repeated switches, provided that these are appropriately managed and supported by clear guidelines [24,25] (Table 4).

## 3. Discussion

The results of this study demonstrate that, within a relatively brief period (2022–2024), there has been a progressive increase in the adoption of biosimilar drugs across various therapeutic areas. This trend is particularly marked for certain high-cost drugs—including bevacizumab, rituximab, infliximab, and trastuzumab—where the market share of the corresponding biosimilar versions has reached levels approaching or exceeding 90%. In contrast, in other fields—especially in insulin therapy and in the case of certain drugs used for autoimmune diseases (adalimumab and etanercept)—the transition to biosimilars, although steadily growing, remains incomplete. A primary point of discussion concerns the differential penetration of biosimilars depending on the therapeutic area. On the one hand, molecules with well-established efficacy and safety in oncology and hematology have been more rapidly embraced by healthcare professionals; this likely reflects the availability of robust scientific evidence derived from both extensive registration studies and real-world clinical experience that confirms the therapeutic equivalence of biosimilars relative to their originators [26]. On the other hand, in certain therapeutic categories, resistance factors persist, attributable both to prescribers’ limited familiarity with the new biosimilars and to logistical and procedural issues related to procurement. A similar variability is observed in therapies for autoimmune and rheumatologic conditions. Although the data confirm a steady increase in the proportion of biosimilar usage (for example, for adalimumab and etanercept), several obstacles still hinder a complete transition to these more economically sustainable drugs. In some cases, temporary shortages in biosimilar availability—due to production or distribution issues—have led physicians to revert to the originator or to initiate new tenders, thereby generating reverse switches and impeding the continuous adoption of biosimilars. Within this context, regional health policies and awareness programs promoted both at the regional level and by the ATS assume particular significance, aiming to reduce costs and promote the rational use of resources. The observed increase in biosimilar prescriptions among naïve patients between 2023 and 2024 suggests that educational interventions targeted at healthcare professionals—coupled with informational campaigns for patients—can enhance confidence in these drugs and help overcome persistent cultural barriers. A crucial aspect emerging from the data concerns the magnitude of the savings already achieved (or potentially achievable) through a broader adoption of biosimilars. The estimate of a “complete switch” to biosimilars—considering all off-patent molecules included in the analysis—indicates a potential total saving of approximately EUR 7.17 million in 2022, EUR 6.21 million in 2023, and a notable EUR 23.54 million in 2024. The significant increase in the latter figure is largely driven by the market introduction during 2023 and 2024 of biosimilars for five high-cost drugs (eculizumab, natalizumab, ranibizumab, tocilizumab, and ustekinumab). Specifically, each of these molecules was launched on different dates (eculizumab in August 2023, natalizumab in July 2024, ranibizumab in October 2023, tocilizumab in December 2023, and ustekinumab in June 2024); consequently, the market penetration data for 2024 remain limited. However, these active substances are characterized by a particularly high annual expenditure for the National Health Service, which in some cases exceeds EUR 7 million per year for a single drug (for instance, eculizumab and natalizumab). Therefore, the expanded use of their respective biosimilar versions may in the future ensure further cost containment, estimated at over EUR 19 million (i.e., the difference between the total EUR 23.54 million and the EUR 4.20 million potential savings calculated by excluding the five aforementioned molecules). In the near future, the implementation of biosimilars for eculizumab, natalizumab, ranibizumab, tocilizumab, and ustekinumab will represent a strategic priority, given their expenditure profiles and the growing body of scientific evidence supporting their safety and efficacy. From a broader perspective, the results corroborate international findings that a structured utilization of biosimilars leads to significant expenditure savings, which can be reinvested to support pharmaceutical innovation and other sectors of healthcare [27]. In the Italian context, the role of regulatory authorities and health protection agencies is crucial: systematic monitoring, the establishment of quantitative penetration targets, and the periodic updating of prescribing guidelines represent effective instruments for promoting the appropriate use of biosimilars while safeguarding the sustainability of the National Health Service. The observed variability in the occurrence of “switches” (both from biosimilar to originator and vice versa), as well as in cases of “multi-switch” and “swap” between different biosimilars, reflects the dynamic nature of an ever-evolving pharmaceutical market and underscores the importance of diversified therapeutic strategies. Robust evidence in the literature has demonstrated that patient clinical stability is maintained despite transitions between different formulations [28,29,30]. These findings confirm that, when managed according to shared protocols, switching does not represent an impediment to effective care. Possible directions for future research include the following:

- An in-depth analysis of the long-term clinical–economic impact: Prospective and retrospective real-world evidence studies should be planned to more precisely measure treatment duration, therapeutic adherence, the number of switches, and potential benefits in terms of clinical outcomes. This would provide more robust data to reinforce the confidence of clinicians and patients.
- A possible revision of procurement procedures: To foster the use of biosimilars, it is advisable to refine regional tenders and procurement contracts, in order to reward not only the lowest price but also supply continuity and long-term stability. This strategy would mitigate the risk of sudden shortages and reduce the occurrence of reverse switches.
- An evaluation of the effectiveness of training and awareness campaigns: Quantifying the impact of various educational initiatives (targeted at both specialist and general practitioners as well as hospital pharmacists) and informational campaigns (addressed to patients) on biosimilar adoption levels.

In summary, the results underscore the growing role of biosimilars as a key instrument in promoting the sustainability and accessibility of biological therapies. Despite heterogeneous adoption rates and challenges encountered in certain therapeutic areas, the trend is clearly oriented towards an increased utilization of these drugs, in line with scientific evidence, regulatory recommendations, and public cost containment objectives. Future studies, supported by rigorous monitoring of real-world data, may offer further insights for developing increasingly effective pharmaceutical governance strategies, benefiting both patients and the entire healthcare system.

## 4. Materials and Methods

During the three-year period from 2022 to 2024, a comprehensive analysis was conducted on the dispensation of pharmacological therapies utilizing biologic drugs with expired patents. This study encompassed all dispensations from hospital pharmacies of both public and private healthcare institutions accredited by the National Health Service and operating within the ATS Milan jurisdiction. The raw data, of an administrative and accounting nature, were sourced from individual data streams produced by each healthcare facility and subsequently transmitted to regional offices for the reimbursement of the administered treatments, comprising a total of 4,970,685 initial records. Specifically, the analysis considered twenty active ingredients for which, since 2022, at least one biosimilar has been commercially available in Italy (Table 5).

For five drugs—Natalizumab, Eculizumab, Ustekinumab, Tocilizumab, and Ranibizumab—the analysis was performed separately, as their market introduction in Italy occurred after 2022 (namely, in July 2024, August 2023, June 2024, December 2023, and October 2023, respectively). Each record extracted from the regional data streams represented a single dispensation to an individual patient and included, at a minimum, the following details: a unique patient identifier, description of the active ingredient, trade name, formulation, ATC code, dispensation date, unit of measure, dispensed quantity, and the corresponding cost. These raw regional data were consolidated into a Microsoft Access 2019 database (Microsoft Corporation, Redmond, WA, USA), which was extensively employed to integrate, organize, and manage the large volume of administrative and accounting information. Consistency checks and verification procedures were performed on the collected data prior to their export to Microsoft Office Excel 2019 (Microsoft Corporation, Redmond, WA, USA). To ensure patient anonymity, no personal demographic data were used; instead, each patient was assigned an automatically generated unique alphanumeric code. One notable challenge encountered pertained to the heterogeneity of units of measure, as healthcare facilities employed various modes of expression (e.g., packages, pieces, mg, mL). Consequently, each value was converted into the “number of posologic units” (defined as a single piece) through a normalization process. Normalization coefficients were computed to convert various measurement units into this unified unit. Conversion factors provided in the formulation details were applied; for example, quantities reported as “packs” were converted into individual pieces by multiplying by the number of pieces per pack, while measurements in mg or mL were converted using the corresponding factors. This approach ensured that each dispensed quantity, expressed as the ratio between the actual amount and the standard dosage defined for that formulation, was consistently comparable across heterogeneous data formats. The initial dataset consisted of 4,970,685 raw records, which included all drug dispensations reported over the three-year period (2022–2024) by healthcare facilities under the jurisdiction of ATS Milano. From this comprehensive dataset, a subset of 418,937 records was identified, corresponding exclusively to the twenty active substances listed in Table 5, each of which had at least one biosimilar commercially available in Italy during the study period. The data cleaning process involved several key steps to ensure analytical accuracy and consistency. First, exclusion criteria were applied to remove records unrelated to the biosimilar-active ingredients under study. Then, all entries with missing critical fields (e.g., active ingredient, quantity, cost) were discarded. Outliers were detected and excluded based on implausible values (e.g., negative quantities or costs, or quantities exceeding reasonable therapeutic ranges), using interquartile range (IQR)-based thresholds and manual cross-verification when necessary. Subsequently, the records pertaining to the drugs under analysis (a total of 418,937 records) were exported from the Microsoft Access 2019 database and transferred to Microsoft Office Excel 2019 (Microsoft Corporation, Redmond, WA, USA), where the analytical process continued. In this phase, the data were further processed using macros developed in Microsoft Visual Basic, Applications Edition (VBA), pivot tables, and advanced formulas. While the use of Microsoft Excel and VBA offers flexibility, it may introduce potential limitations such as manual handling errors and constraints in processing very large datasets. These limitations were mitigated by implementing a double-check procedure, wherein a sample of the Excel outputs was validated against the original Access database using automated scripts, and cross-validation with independent data extracts was conducted to promptly identify and correct any discrepancies. Finally, a comparative analysis was performed on the type of drug (originator or biosimilar) administered to treatment-naïve patients during 2023 and 2024, with 2022 serving as the reference year for cohort definition. In addition, patients who underwent therapeutic switches for the same active ingredient over the three-year period were identified, including transitions from biosimilar to originator, from originator to biosimilar, and instances of multiswitching among biosimilars. For the five active ingredients whose biosimilars were introduced after 2022, data were analyzed separately in a subsequent phase, while annual figures were still reported to capture early trends in biosimilar market penetration.

## 5. Conclusions

The issue of biosimilars has emerged as a focal point for several key priorities in healthcare policy, including regional governance, sustainability, cost containment, and prescribing autonomy [31,32,33]. It is now widely acknowledged that pharmacological therapies—particularly innovative treatments based on biological drugs—impact multiple facets of both clinical practice and care delivery, thereby necessitating strategic coherence in decisions that drive healthcare system innovation. In this context, the Lombardy Region distinguishes itself as an exemplary model of public healthcare management, owing to policy orientations designed to reconcile fiscal equilibrium with care efficiency. Concurrently, institutional and organizational strategies must be complemented by appropriate adjustments in procurement policies. Indeed, the availability of the drug constitutes the indispensable basis for any policy decision: while economic considerations are crucial, they cannot be regarded as the sole evaluative parameter. The implementation of modern procurement tools enables the simultaneous assurance of drug availability, prescribing autonomy and appropriateness, along with economic savings. This approach is realized through the active engagement of general practitioners and hospital pharmacies, which are pivotal in a complex yet effective governance system. The adoption of biosimilars, examined over the 2022–2024 triennium, emerges as the result of a multifaceted dynamic in which clinical, economic, organizational, and governance factors converge in shaping therapeutic decisions. Documented evidence suggests that in settings marked by robust scientific validation and economic benefits, the use of biosimilars can reach significantly high levels; concurrently, challenges persist in areas where entrenched prescribing practices and procurement issues limit their utilization. These results provide critical insights for steering future healthcare policy strategies, aimed at ensuring an increasingly sustainable and efficient care system capable of addressing the needs of a continuously evolving patient population. Moreover, the disparities observed in biosimilar adoption rates across various therapeutic classes highlight concrete opportunities to optimize the healthcare system and enhance access to cost-effective treatments [34,35]. Strategic initiatives, both at the regional level and within healthcare facilities operating under ATS Milano, have fostered deliberate and informed use of biosimilars, grounded in demonstrated clinical equivalence and the potential for significant economic savings. These initiatives, which encompassed continuous monitoring of prescribing patterns, targeted educational campaigns, and direct communication with prescribers and strategic leadership, have substantially contributed to overcoming preconceived barriers to the adoption of these medications. Finally, the significant increase in the use of biosimilars, as documented during the 2022–2024 period, represents a fundamental milestone in the optimization of biological therapies. Although significant progress has been achieved, certain areas remain underutilized, necessitating targeted interventions. The success of the monitoring and promotional initiatives implemented, along with the findings from the analysis of the 2022–2024 triennium, not only attests to the transformative potential of biosimilars in healthcare but also provides a replicable model for other regions and systems, thereby contributing to the attainment of sustainable and equitable health outcomes.

## Figures and Tables

**Table 1:** Incidence of biosimilar drug use in the years of analysis (2022, 2023, and 2024). The columns report the total quantity of drug dispensed for each active ingredient under examination, the number of biosimilar dosage units, and the percentage of biosimilar use over the total.

Drug Name	2022			2023			2024		
	Total Units	Biosim. (N.)	Biosim. (%)	Total Units	Biosim. (N.)	Biosim. (%)	Total Units	Biosim. (N.)	Biosim. (%)
Adalimumab	91,145	65,401	71.8%	101,718	78,993	77.7%	108,714	93,775	86.3%
Bevacizumab	14,686	13,478	91.8%	11,976	11,786	98.4%	11,300	11,258	99.6%
Eculizumab *	1943	0	0.0%	2389	0	0.0%	2756	204	7.4%
Enoxaparin Sodium	87,657	4302	4.9%	89,643	17,747	19.8%	85,007	48,766	57.4%
Epoetin Alfa	5834	2900	49.7%	6039	3437	56.9%	6435	3933	61.1%
Epoetin Zeta	5807	5807	100.0%	5986	5986	100.0%	4359	4359	100.0%
Etanercept	62,144	36,784	59.2%	63,376	40,158	63.4%	64,336	47,480	73.8%
Filgrastim	13,878	12,433	89.6%	14,737	13,286	90.2%	12,483	10,956	87.8%
Infliximab	34,458	31,892	92.6%	40,915	38,924	95.1%	43,743	42,420	97.0%
Insulin Aspart	22,749	0	0.0%	23,543	0	0.0%	20,599	0	0.0%
Insulin Glargine	757	276	36.5%	824	220	26.7%	1074	188	17.5%
Insulin Lispro	144	0	0.0%	207	0	0.0%	320	5	1.6%
Natalizumab *	5648	0	0.0%	6059	0	0.0%	9013	443	4.9%
Drug name		2022			2023			2024	
	Total units	Biosim. (N.)	Biosim. (%)	Total units	Biosim. (N.)	Biosim. (%)	Total units	Biosim. (N.)	Biosim. (%)
Pegfilgrastim	1982	1981	99.9%	2454	2445	99.6%	2518	2518	100.0%
Ranibizumab *	13,983	0	0.0%	12,683	0	0.0%	8321	1161	14.0%
Rituximab	11,278	10,752	95.3%	12,258	11,813	96.4%	12,290	12,032	97.9%
Teriparatide	149	8	5.4%	170	11	6.5%	145	84	57.9%
Tocilizumab *	31,462	0	0.0%	35,179	0	0.0%	40,815	1772	4.3%
Trastuzumab	17,151	16,964	98.9%	13,283	13,204	99.4%	11,216	11,165	99.5%
Ustekinumab *	6336	0	0.0%	8171	0	0.0%	8929	269	3.0%

* Biosimilars for the following drugs became commercially available on the indicated dates: Eculizumab—August 2023, Natalizumab—July 2024, Ranibizumab—October 2023, Tocilizumab—December 2023, Ustekinumab—June 2024.

**Table 2:** Number of naïve patients who initiated therapy with a biosimilar or the originator for each listed biologic agent in 2023 and 2024. Data are presented by drug, with separate columns indicating the number of patients started on a biosimilar versus the originator in each year.

Drug	Naïve Bios. 2023	Naïve Orig. 2023	Naïve Bios. 2024	Naïve Orig. 2024
Adalimumab	1050	124	1127	46
Bevacizumab	510	9	512	3
Eculizumab *	0	18	1	18
Enoxaparin Sodium	779	5066	2534	2356
Epoetin Alfa	69	48	83	31
Epoetin Zeta	115	0	95	0
Etanercept	268	27	293	16
Filgrastim	760	71	588	91
Infliximab	445	5	407	6
Insulin Aspart	0	40	0	14
Insulin Glargine	32	2	27	5
Insulin Lispro	0	2	0	2
Natalizumab *	0	134	18	113
Pegfilgrastim	541	5	580	0
Ranibizumab *	0	1516	112	871
Rituximab	1156	30	1136	14
Teriparatide	0	3	2	2
Tocilizumab *	0	282	27	322
Trastuzumab	508	0	552	0
Ustekinumab *	0	317	14	320

* Biosimilars for the following drugs became commercially available on the indicated dates: Eculizumab—August 2023, Natalizumab—July 2024, Ranibizumab—October 2023, Tocilizumab—December 2023, Ustekinumab—June 2024.

**Table 3:** Annual expenditure (in EUR) for biosimilars and originators by drug name, for the years 2022, 2023, and 2024.

Drug	Biosimilar Expenditure 2022 (EUR)	Biosimilar Expenditure 2023 (EUR)	Biosimilar Expenditure 2024 (EUR)	Originator Expenditure 2022 (EUR)	Originator Expenditure 2023 (EUR)	Originator Expenditure 2024 (EUR)
Adalimumab	2,900,790	3,423,767	3,698,916	4,026,478	3,745,818	2,446,345
Bevacizumab	1,921,223	1,601,312	1,153,617	610,304	86,626	24,663
Eculizumab *	-	-	364,113		7,934,768	8,246,430
Enoxaparin Sodium	5649	28,407	69,684	118,598	108,672	51,960
Epoetin Alfa	189,984	249,149	342,561	351,836	323,053	309,517
Epoetin Zeta	167,715	175,714	116,484	-	-	-
Etanercept	1,753,022	1,945,104	2,464,618	3,813,805	3,514,185	2,482,370
Filgrastim	59,137	69,327	58,143	93,781	101,731	108,681
Infliximab	3,088,609	3,950,822	4,435,347	741,299	575,186	382,205
Insulin Aspart	-	-	-	75,738	64,558	56,760
Insulin Glargine	1939	1546	1320	12,705	16,388	19,040
Insulin Lispro	-	-	26	458	631	1120
Natalizumab *	-	-	425,574		8,247,294	7,304,026
Pegfilgrastim	162,197	182,865	157,532	685	6165	
Ranibizumab *	-	-	268,813	6,913,999	5,039,293	2,729,022
Rituximab	1,870,662	1,682,269	1,464,047	442,939	352,357	229,145
Teriparatide	1628	2176	13,855	47,026	43,014	12,901
Tocilizumab *	-	-	255,572	6,319,237	6,466,113	6,552,750
Trastuzumab	2,109,615	1,200,323	934,065	63,323	26,556	17,278
Ustekinumab *	-	-	197,201	14,315,206	18,407,717	19,304,575

* Biosimilars for the following drugs became commercially available on the indicated dates: Eculizumab—August 2023, Natalizumab—July 2024, Ranibizumab—October 2023, Tocilizumab—December 2023, Ustekinumab—June 2024.

**Table 4:** Number of switches (biosimilar → originator and originator → biosimilar), swaps (biosimilar → biosimilar), and multi-switches recorded for major biologic drugs and their respective biosimilars from 2022 to 2024.

Drug	Switch Biosim. → Orig.			Switch Orig. → Biosim.			Swap Biosim. → Biosim.			Multi-Switch		
	2022	2023	2024	2022	2023	2024	2022	2023	2024	2022	2023	2024
Adalimumab	11	10	7	68	95	312	28	487	301	69	45	53
Bevacizumab	1	0	0	55	1	6	1	79	78	20	7	1
Eculizumab *	0	0	0	0	0	9	0	0	0	0	0	0
Enoxaparin Sodium	2	7	18	0	81	196	0	3	10	5	6	17
Epoetin Alfa	8	23	8	2	1	3	0	0	0	23	28	22
Epoetin Zeta	0	0	0	0	0	0	0	0	0	0	0	0
Etanercept	3	27	6	12	35	98	5	85	118	121	135	37
Filgrastim	3	11	30	5	7	27	70	262	82	20	33	47
Infliximab	1	0	0	16	6	38	119	242	245	8	4	6
Insulin Aspart	0	0	0	0	0	0	0	0	0	0	0	0
Insulin Glargine	0	0	0	0	0	0	0	0	0	1	1	1
Insulin Lispro	0	0	0	0	0	0	0	0	0	0	0	0
Natalizumab *	0	0	0	0	0	104	0	0	0	0	0	8
Pegfilgrastim	0	0	0	0	2	1	13	5	8	0	0	0
Ranibizumab *	0	0	0	0	0	375	0	0	0	0	0	3
Rituximab	3	2	2	3	10	11	169	5	337	7	8	2
Teriparatide	0	0	0	0	1	4	0	0	0	0	0	1
Tocilizumab *	0	0	0	0	0	26	0	0	0	19	15	54
Trastuzumab	1	0	0	4	0	0	397	168	72	19	7	10
Ustekinumab *	0	0	0	0	0	213	0	0	0	0	0	0

* Biosimilars for the following drugs became commercially available on the indicated dates: Eculizumab—August 2023, Natalizumab—July 2024, Ranibizumab—October 2023, Tocilizumab—December 2023, Ustekinumab—June 2024.

**Table 5:** Drugs under study and analysis period based on the year of biosimilar commercialization.

ATC	Drugs	Analysis Period
A10AB04	Insulin Lispro	2022–2024
A10AB05	Insulin Aspart	2022–2024
A10AE04	Insulin Glargine	2022–2024
B01AB05	Enoxaparin Sodium	2022–2024
B03XA01	Epoetin Alfa	2022–2024
B03XA01	Epoetin Zeta	2022–2024
H05AA02	Teriparatide	2022–2024
L01XC02	Rituximab	2022–2024
L01XC03	Trastuzumab	2022–2024
L01XC07	Bevacizumab	2022–2024
L03AA02	Filgrastim	2022–2024
L03AA13	Pegfilgrastim	2022–2024
L04AA23	Natalizumab	2024
L04AA06	Eculizumab	2023–2024
L04AB01	Etanercept	2022–2024
L04AB02	Infliximab	2022–2024
L04AB04	Adalimumab	2022–2024
L04AC05	Ustekinumab	2024
L04AC07	Tocilizumab	2023–2024
S01LA04	Ranibizumab	2023–2024

## Data Availability

Data are available upon reasonable request.

## Abbreviations

AIFA	Italian Medicines Agency
ASST	Territorial Healthcare and Social Agencies
ATS	Health Protection Agencies
EMA	European Medicines Agency
HMA	Heads of Medicines Agencies
IRCCS	Scientific Institutes for Research, Hospitalization and Healthcare
